# Geriatric Trauma Outcome Score as a Mortality Predictor in Isolated Moderate to Severe Traumatic Brain Injury: A Single-Center Retrospective Study

**DOI:** 10.3390/healthcare12161680

**Published:** 2024-08-22

**Authors:** Ching-Ya Huang, Yuan-Hao Yen, Ching-Hua Tsai, Shiun-Yuan Hsu, Po-Lun Tsai, Ching-Hua Hsieh

**Affiliations:** 1 Department of Plastic Surgery, Kaohsiung Chang Gung Memorial Hospital, Chang Gung University College of Medicine, Kaohsiung 83301, Taiwan; b101106030@tmu.edu.tw (C.-Y.H.); medik@cgmh.org.tw (Y.-H.Y.); 2 Department of Trauma Surgery, Kaohsiung Chang Gung Memorial Hospital, Chang Gung University College of Medicine, Kaohsiung 83301, Taiwan; tsai1737@cloud.cgmh.org.tw (C.-H.T.); ah.lucy@hotmail.com (S.-Y.H.); 3 Department of Plastic Surgery, Chiayi Chang Gung Memorial Hospital, Chang Gung University College of Medicine, Chiayi 61363, Taiwan

**Keywords:** trauma, Abbreviated Injury Scale (AIS), traumatic brain injury (TBI), Geriatric Trauma Outcome Score (GTOS), mortality

## Abstract

Background: Traumatic brain injury (TBI) is a major cause of mortality and disability worldwide, with severe cases significantly increasing the risk of complications and long-term mortality. The Geriatric Trauma Outcome Score (GTOS), based on age, injury severity, and transfusion need, has been validated for predicting mortality in older trauma patients, but its utility in predicting mortality for TBI patients remains unexplored. Methods: This retrospective study included 5543 adult trauma patients with isolated moderate to severe TBI, defined by head Abbreviated Injury Scale (AIS) scores of ≥ 3, from 1998 to 2021. GTOS was calculated with the following formula: age + (Injury Severity Score × 2.5) + 22 (if transfused within 24 h). The area under the receiver operating characteristic curve (AUROC) assessed GTOS’s ability to predict mortality. The optimal GTOS cutoff value was determined using Youden’s index. Mortality rates were compared between high- and low-GTOS groups, separated by the optimal GTOS cutoff value, including a propensity score-matched analysis adjusting for baseline characteristics. Results: Among 5543 patients, mortality was 8.3% (462 deaths). Higher mortality is correlated with male sex, older age, higher GTOS, and comorbidities like hypertension, coronary artery disease, and end-stage renal disease. The optimal GTOS cut-off for mortality prediction was 121.5 (AUC = 0.813). Even when the study population was matched by propensity score, patients with GTOS ≥121.5 had much higher odds of death (odds ratio 2.64, 95% confidence interval 1.93–3.61, *p* < 0.001) and longer hospital stays (mean 16.7 vs. 12.2 days, *p* < 0.001) than those with GTOS < 121.5. Conclusions: These findings support the idea that GTOS is a useful tool for risk stratification of in-hospital mortality in isolated moderate to severe TBI patients. However, we encourage further research to refine GTOS for better applicability in TBI patients.

## 1. Introduction

Traumatic brain injury (TBI) is a major cause of death and disability worldwide, with severe cases significantly elevating the risk of mortality due to various complications. Researchers have found that TBI patients have a 1.5 times higher likelihood of death over a four-decade period [1]. Individuals with severe TBI are at a higher risk of death from circulatory conditions, pneumonia, and aspiration pneumonia compared to the general population [2]. TBI significantly increases mortality rates, with factors such as age, lower Glasgow Coma Scale (GCS) scores, higher injury severity, and specific injury types serving as critical predictors of outcomes [3]. For instance, older population, although representing a smaller percentage of TBI cases, account for a substantial proportion of long-term mortality [4]. Thus, early recognition of risk factors associated with mortality in TBI patients is crucial for implementing timely and effective interventions and reducing long-term mortality rates.

Older populations, generally those aged 65 and above, are particularly vulnerable due to higher rates of comorbidities, reduced physiological reserves, and an increased risk of complications from injuries. With the aging global population, the incidence of trauma among the older population is on the rise, posing significant challenges in medical management and outcome prediction. The Geriatric Trauma Outcome Score (GTOS) was developed to address the complexities associated with aging, which incorporates age, injury severity, and the need for blood transfusions within the first 24 h and has shown a strong capability to predict mortality in older population with varying injury severities [4,5,6,7,8,9]. The GTOS formula is as follows: age + Injury Severity Score (ISS) × 2.5 + 22 (if any packed red blood cells were transfused within 24 h of admission). It also suggests that GTOS is beneficial for predicting mortality among older trauma patients and for predicting short- and long-term survival [6,10,11], as well as in decisions regarding invasive or aggressive treatments [7,8]. Additionally, the simplicity of GTOS contrasts with more complex models like CRASH and IMPACT, which incorporate detailed neurological assessments, such as the GCS, pupil reactivity, and image findings, to predict outcomes in TBI patients. While GTOS demonstrates good predictive accuracy for in-hospital mortality, especially in the older population [12,13], the CRASH and IMPACT models offer broader applicability with high predictive values, depending on the specific outcome and population [14]. These traditional TBI models are particularly useful for predicting both short-term and long-term outcomes, making them suitable for diverse TBI populations. Ultimately, GTOS excels in its ease of use, whereas CRASH and IMPACT provide more comprehensive risk stratification in TBI cases. Nonetheless, the GTOS may aid in the decision-making process for treating TBI patients, assisting physicians and families in making more informed treatment decisions.

Overall, prognostic scoring systems are extensively studied for classifying trauma patients with critical illness [15,16,17,18]. However, these systems often require substantial data or certain baseline characteristics, which might be difficult to get promptly. The complexities of TBI patients necessitate a quick prognostic method capable of detecting minor changes in patient status and providing real-time insights critical for dynamic decision-making and optimal management. In addition, the GTOS, which was originally created for older people owing to its rapid computation, has not been validated for TBI patients. Therefore, the purpose of this study is to assess the GTOS’s usefulness as a quick and accurate predictive tool for in-hospital mortality risk and risk stratification in TBI patients in critical care settings.

## 2. Materials and Methods

### 2.1. Patient Enrollment and Study Design

The procedure was approved by Chang Gung Memorial Hospital’s Institutional Review Board (IRB) prior to the start of the research under approval number 202400775B0. Because this study is retrospective in nature, patient permission was not required. This study examined registered medical data from 1 January 1998 to 31 December 2021 from the Trauma Registry System in a Level I trauma center in southern Taiwan [19]. The trauma database was prospectively maintained by two full-time, qualified registrants with nursing backgrounds, who entered each hospitalized trauma patient’s registered data into the hospital trauma registry following validation by a trauma surgeon. The study includes all patients aged equal to or over 20 who had isolated moderate to severe TBI, defined by head Abbreviated Injury Scale (AIS) ≥ 3. Exclusion criteria include patients with burns, hangings, and those with trauma that had AIS ≥ 3 in other body regions. The research method involves detailed recording of all retrieved cases’ sex, age, blood transfusion history, GTOS, comorbidities such as cerebrovascular accident (CVA), hypertension (HTN), coronary artery disease (CAD), congestive heart failure (CHF), diabetes mellitus (DM), end-stage renal disease (ESRD), GCS, Injury Severity Score (ISS), in-hospital mortality, and hospital stay. The GTOS is calculated by adding the age to the product of ISS multiplied by 2.5, and then adding 22 if any packed red blood cells were transfused within 24 h of arrival. The values in the GTOS formula, such as the 2.5 multiplier for ISS and the addition of 22 points for transfusion within 24 h, were derived from regression analyses to optimize mortality prediction in geriatric trauma patients. The 2.5 multiplier enhances sensitivity to injury severity, while the 22-point addition reflects the significant mortality risk associated with early transfusion, indicating severe trauma [7].

### 2.2. Statistical Analysis

The statistical analysis was conducted with the assumption that different levels of GTOS are related to varying in-hospital mortality risks in TBI patients. The chi-square test was used to compare the proportions of categorical variables between the deceased and the survivors, as well as to calculate the odds ratio (OR) and 95% confidence interval (CI). Levene’s test was used to ensure that variances were homogeneous, followed by an analysis of variance (ANOVA) to assess differences in continuous variables across groups. The Mann-Whitney U test was used for non-normally distributed continuous variables, which presented as the median and interquartile range (IQR). Univariate and multivariate analyses were carried out to determine the independent risk factors related to in-hospital mortality. The area under the receiver operating characteristic curve (AUROC) was used to forecast in-hospital mortality performance and define the optimal cut-off value for the GTOS, which was computed using the Youden index [20,21]. The risk of in-hospital mortality was also determined after correcting for potential confounding factors such as age, sex, comorbidities, and conscious level using a propensity-score-matched patient cohort analysis. Statistical analyses were carried out with IBM SPSS Statistics for Windows, Version 23. A *p*-value of <0.05 was considered statistically significant.

## 3. Results

### 3.1. Enrollment of the Patients

**Figure 1:**
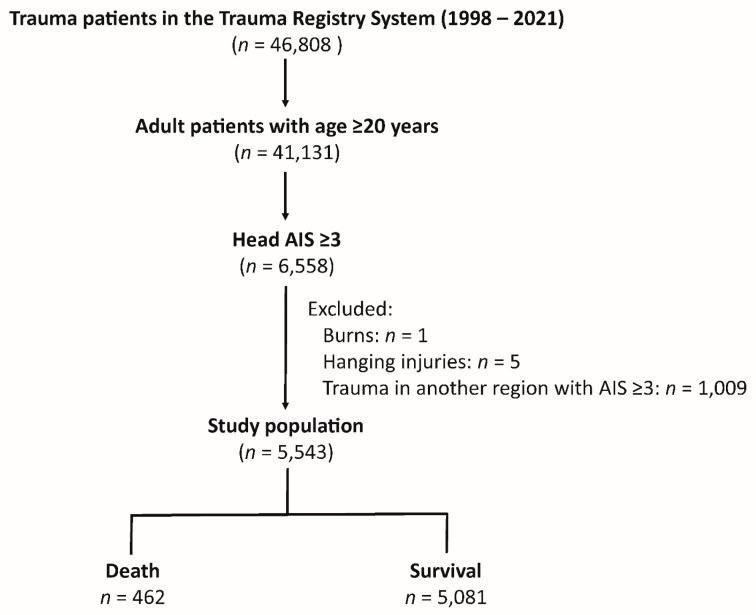
Patient enrollment flowchart.

The study cohort included 46,808 trauma patients in the Trauma Registry System from 1998 to 2021 (Figure 1). Among these, 41,131 were adult patients (age ≥ 20). Out of these, 6558 patients had head AIS (Abbreviated Injury Scale) scores of ≥ 3. The study further excluded patients with burns (*n* = 1), hanging injuries (*n* = 5), and trauma to other body regions that also had AIS scores ≥ 3 (*n* = 1009). There were no patients with missing data who would be excluded from this study. This resulted in a final study population of 5543 patients who had an isolated moderate to severe TBI. Within this cohort, there were 462 deaths, and 5081 patients survived.

### 3.2. Demographic and Clinical Characteristics of Patients Stratified by in-Hospital Mortality Status

Table 1 presents the demographic characteristics and clinical variables for the study population. Patients who died had significantly higher odds of being male (67.5% vs. 61.1%, *p* = 0.006) and were generally older (mean age 64.1 vs. 58.0 years, *p* < 0.001). They required more blood transfusions (mean 2.4 vs. 0.4 units, *p* < 0.001) and had higher GTOS scores (mean 131.2 vs. 100.5, *p* < 0.001). The prevalence of certain comorbidities was also higher among deceased patients, including HTN (42.9% vs. 36.4%, *p* = 0.006), CAD (12.6% vs. 6.3%, *p* < 0.001), and ESRD (10.2% vs. 2.5%, *p* < 0.001). Deceased patients had significantly lower median GCS scores (median 5 vs. 15, *p* < 0.001), with a higher percentage having scores in the 3–8 range (69.0% vs. 12.9%, *p* < 0.001). They also had higher ISS (median 25 vs. 16, *p* < 0.001), with a greater proportion having ISS ≥25 (66.9% vs. 8.4%, *p* < 0.001). Lastly, the length of hospital stay was shorter for deceased patients compared to survivors (mean 9.3 vs. 12.4 days, *p* < 0.001).

### 3.3. Univariate and Multivariate Analysis of Factors Associated with In-Hospital Mortality in Isolated Moderate to Severe TBI Patients

In Table 2, the univariate analysis revealed that male sex (OR 1.33, *p* = 0.007), older age (OR 1.19, *p* < 0.001), higher GTOS scores (OR 1.67, *p* < 0.001), and the presence of comorbidities such as HTN (OR 1.31, *p* = 0.006), CAD (OR 2.14, *p* < 0.001), and ESRD (OR 4.45, *p* < 0.001) were significantly associated with increased in-hospital mortality. A higher ISS (OR 1.26, *p* < 0.001) was also linked to increased in-hospital mortality. In the multivariate analysis, being male (OR 1.46, *p* = 0.002), having a higher GTOS score (OR 1.33, *p* < 0.001), having ESRD (OR 4.72, *p* < 0.001), and having a higher ISS (OR 1.17, *p* < 0.001) were still strong predictors of death. A strong collinearity of GTOS with age and ISS was expected in the analysis of variance inflation factor (VIF) in the multivariate analysis (Table 3), seeing as GTOS is calculated based on age and ISS. However, male gender, HTN, CAD, and ESRD have low VIF values, indicating minimal collinearity with other predictors.

### 3.4. The Optimal Cut-Off Value of GTOS in Predicting In-Hospital Mortality

**Figure 2:**
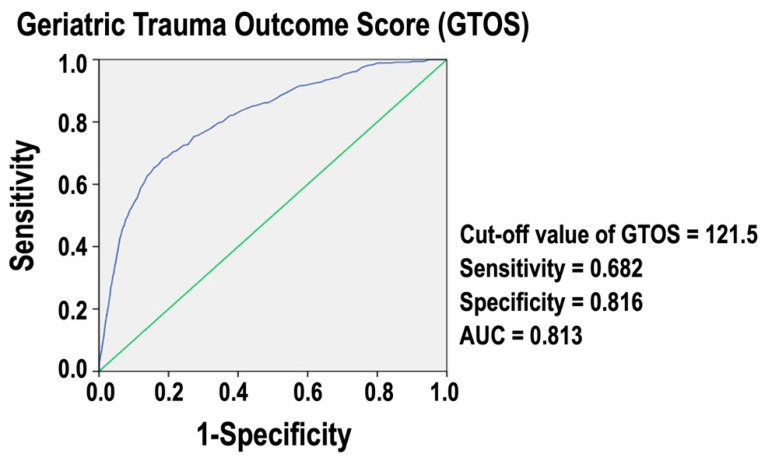
ROC curve for GTOS predicting in-hospital mortality of isolated moderate to severe TBI patients.

Figure 2 presents a ROC curve for GTOS. The analysis identifies an optimal cut-off value of 121.5 for predicting in-hospital mortality. At this threshold, the sensitivity is 0.682, and the specificity is 0.816. The AUC is 0.813, indicating that GTOS is an accurate measure for assessing in-hospital mortality risk in these trauma patients. An AUC value close to 1 denotes excellent predictive power, while an AUC value of 0.5 would suggest no predictive ability. This result demonstrates that the GTOS is a reliable tool for predicting in-hospital mortality in the isolated moderate to severe TBI patients.

### 3.5. Comparative Demographics Outcomes Based on Grouping by GTOS

Table 4 compares the demographics and clinical outcomes of patients with high (≥121.5) versus low (<121.5) GTOS scores. We chose the threshold value of GTOS of 121.5 based on the optimal cut-off value of GTOS for predicting in-hospital mortality in the ROC curve analysis. Patients with a high GTOS were significantly older (mean age 73.6 vs. 54.1 years, *p* < 0.001), required more blood transfusions (mean 1.8 vs. 0.2 units, *p* < 0.001), and had a higher prevalence of comorbidities such as CVA, HTN, CAD, CHF, DM, and ESRD. Patients in the high-GTOS group had lower median GCS scores (median 13 vs. 15, *p* < 0.001), a higher percentage having scores in the range of 3–8 (34.6% vs. 12.6%, *p* < 0.001), and a higher ISS (median 24 vs. 16, *p* < 0.001), with a greater proportion having ISS ≥ 25 (45.0% vs. 4.1%, *p* < 0.001). In-hospital mortality was significantly higher in the high-GTOS group (25.2% vs. 3.4%, *p* < 0.001), and their hospital stay was longer (mean 15.7 vs. 11.0 days, *p* < 0.001).

### 3.6. Propensity-Score-Matched Analysis of High and Low GTOS Score Groups

As shown in Table 5, the propensity-score-matched analysis compares patients with high (≥121.5) and low (<121.5) GTOS scores, each group containing 794 patients. There were no significant differences in sex, age, or comorbidities between the groups after matching. Despite these similarities, patients with higher GTOS scores had significantly higher in-hospital mortality rates (18.5% vs. 7.9%, *p* < 0.001) and longer hospital stays (mean 16.7 vs. 12.2 days, *p* < 0.001). This indicates that higher GTOS scores are associated with worse outcomes, even when baseline characteristics are similar.

## 4. Discussion

Our study found that higher GTOS scores are a significant independent risk factor for in-hospital mortality in patients with isolated moderate to severe TBI. The ideal GTOS cut-off value of 121.5 was identified with high predictive accuracy (AUC 0.813). Patients with high GTOS exhibited worse results, including increased in-hospital mortality and longer hospital stays, even after propensity-score matching. These findings support the GTOS’s use as a valid tool for measuring in-hospital mortality risk in isolated moderate to severe TBI patients. This study expanded GTOS’s predictive validity in the context of elder trauma patients to include a new trauma patient cohort, allowing for a more comprehensive assessment of the tool’s potential applications in trauma care.

Several studies have validated the GTOS for its predictive accuracy in assessing mortality risk among older trauma patients. Meagher et al. [11] reported GTOS and GTOS II’s predictive performance with AUROCs of 0.674 and 0.678, respectively. Zhao et al. [7] found an AUC of 0.82 for GTOS, which was superior to using age and ISS alone. Ravindranath et al. [5] and Park et al. [13] reported similar AUCs of 0.838 and 0.832, respectively, reinforcing GTOS’s effectiveness across different populations and settings. Cook et al. [9] confirmed GTOS’s robustness across multiple trauma centers with an AUC of 0.86. Åhl et al. [4] found that GTOS effectively predicted in-hospital mortality with an AUC of 0.87, improving to 0.88 after excluding patients with treatment limitations or those discharged to hospice. However, its performance in predicting 1-year mortality was limited, with a high misclassification rate of 17.6%. Our study results align with these findings, demonstrating that deceased patients had higher GTOS scores. We identified an optimal GTOS cut-off value of 121.5, with an AUC of 0.813, indicating good predictive accuracy. Arslan Erduhan et al. [10] highlighted a lower GTOS cutoff point of ≥95 for predicting 30-day mortality in geriatric blunt trauma patients, with a sensitivity of 76% and specificity of 72.27%. In contrast, our study found an optimal GTOS cutoff of 121.5 for predicting overall mortality in a specific but common trauma population, with a lower sensitivity of 68.2% but higher specificity of 81.6%. These results underscore GTOS’s reliability as a prognostic tool, though the optimal cut-off values and specific predictors may vary based on clinical context and patient demographics.

The varying results in the efficacy of the GTOS across studies can be attributed to differences in study populations, methodologies, and clinical settings. For instance, differences in age distribution, comorbidities, and injury severity among patient populations, as well as the specific subsets of trauma patients studied, may lead to variations in reported AUC values. For example, Zhao et al. [7] focused on a specific cohort in a single trauma center with a consistent approach, while Cook et al. [9] conducted a multicenter study with broader patient demographics and varied clinical practices. Methodological factors, such as the inclusion or exclusion of patients with treatment limitations or the focus on short-term versus long-term outcomes, also impact GTOS performance [4,11]. Additionally, differences in trauma care practices, resources, and protocols across various trauma centers can influence outcomes and, consequently, the predictive accuracy of GTOS. Additionally, the timing and criteria for data collection, such as the inclusion of patients with varying levels of trauma severity and the presence of advanced interventions like intracranial pressure monitoring, could lead to differences in the reported efficacy of GTOS across these studies.

On the other hands, the impact of blood transfusion on TBI outcomes has been the subject of research, highlighting the complexity of transfusion practices in this patient population. Several studies suggest that blood transfusions are generally associated with poorer outcomes, including increased mortality and complications [22]. For instance, Warner et al. [23] observed that transfusions in anemic TBI patients led to worse long-term functional outcomes, indicating that transfusion strategies should be carefully targeted and minimized to avoid exacerbating patient conditions. Furthermore, in pediatric populations, transfusions were linked to higher mortality rates and greater dependency post-discharge, reinforcing the need for cautious transfusion practices in TBI management [24]. Additionally, while anemia is independently associated with poor outcomes in TBI, the benefits of transfusions at specific hemoglobin thresholds remain unclear, emphasizing the necessity for refined guidelines to optimize patient care [25]. The study found that patients who required blood transfusions within 24 h of admission had significantly worse outcomes compared to those who did not. The need for transfusion was associated with higher GTOS values, which were, in turn, linked to increased in-hospital mortality. Specifically, patients who died received an average of 2.4 units of transfusion, compared to just 0.4 units in those who survived. These findings highlight the critical importance of considering transfusion needs in the risk stratification and management of trauma patients, particularly those with moderate to severe TBI.

While our results support the use of GTOS in isolated moderate to severe TBI patients, a group of subjects with high risk for mortality, the application of GTOS to all TBI cases is critical given the complexity and variability of TBI outcomes. Existing research indicates that while GTOS has demonstrated utility in predicting outcomes for geriatric trauma patients, its efficacy specifically for TBI patients is less clear. Simplicity is a key strength of GTOS, making it practical for use in emergency and clinical settings where time and resources may be limited. However, this design choice also means that more complex, TBI-specific factors, such as intracranial pressure, the presence of diffuse axonal injury (DAI), or cerebral contusions, were not included in the score. DAI and brain contusions are two critical types of TBI that significantly impact mortality. DAI, caused by shearing forces during rapid head movements, is often associated with immediate coma, severe disability, and high mortality, especially when the brainstem is involved [26]. Contusions, which are focal injuries leading to localized brain damage and hemorrhage, can exacerbate neurological deficits and increase mortality [27]. The prognosis worsens significantly when these injuries are extensive or involve critical areas of the brain, resulting in high rates of fatality [28]. Moreover, one comment highlighted that the GTOS did not significantly outperform traditional TBI-specific models like IMPACT and CRASH in predicting mortality and long-term outcomes for TBI patients [29]. It emphasized the need for caution when applying GTOS to TBI patients, noting that the score’s reliance on factors like age, ISS, and transfusion needs may not fully capture the nuances of TBI pathology and treatment. Another significant limitation of GTOS in the context of TBI is its failure to account for intracranial pressure, a critical factor in the management and prognosis of TBI patients. Elevated intracranial pressure is a well-established predictor of poor outcomes in TBI, as it reflects the severity of brain injury and the potential for secondary brain damage due to increased pressure within the skull. Barea-Mendoza et al. [30] evaluated the applicability of the GTOS for predicting outcomes in TBI patients using data from the Spanish Trauma ICU registry. The study analyzed 5882 ICU admissions, including 1417 geriatric patients, of whom 804 had significant TBI. The GTOS showed no significant difference in predictive ability for TBI patients compared to non-TBI patients (AUC 0.652 vs. 0.627, *p* = 0.65). Furthermore, the predictive performance of GTOS was notably poorer in patients with severe intracranial hypertension requiring advanced interventions, with the lowest AUC of 0.414 observed in patients admitted for organ donation. The study concludes that while GTOS is moderately effective for general geriatric trauma, its predictive power is limited, particularly concerning intracranial hypertension. Overall, the challenge lies in GTOS’s limitations, particularly its lack of consideration for other TBI-specific factors, which are critical determinants of outcomes in TBI cases [29,30]. Consequently, while GTOS offers a streamlined approach to prognostication, it may require further validation or modifications to enhance its accuracy and reliability for TBI patients.

Overall, the GTOS has been shown to be a practical tool for rapid assessment of mortality risk in trauma patients, including those with moderate to severe TBI. Its simplicity, requiring only a few easily obtainable variables, allows for quick calculation and seamless integration into clinical workflows. GTOS may be seamlessly integrated into clinical settings by incorporating it into electronic health record systems, where it can be automatically calculated using patient data such as age, ISS, and transfusion needs. This allows clinicians to quickly identify high-risk patients and make informed decisions regarding their care. Studies have demonstrated that GTOS can effectively guide resource allocation and prioritize care in trauma centers, improving patient outcomes by enabling early intervention and informed decision-making [12]. This practical application underscores GTOS’s value in enhancing clinical efficiency and patient care. Additionally, integrating GTOS with machine learning may greatly enhance real-time risk prediction in trauma care by identifying complex patterns that improve accuracy. Machine learning models have outperformed traditional methods in predicting outcomes like mortality and multi-organ failure, enabling more personalized and timely interventions [31,32]. This integration may provide clinicians with personalized, real-time insights, optimizing trauma care. There are several limitations to this study. First, the retrospective nature of the study inherently introduces potential biases and limits the ability to establish causality. Second, the data is sourced from a single Level I trauma center, which may not be generalizable to other settings or populations. Third, the study relies on the accuracy and completeness of medical records, which can vary and may affect the reliability of the findings. Fourth, the GTOS does not account for certain critical factors specific to TBI, such as intracranial pressure or brain injury types, which are crucial for assessing the severity and prognosis of TBI. Lastly, the study did not include long-term outcomes beyond in-hospital mortality, limiting the understanding of GTOS’s effectiveness in predicting extended survival and functional recovery in TBI patients. Future studies should address these limitations by conducting prospective, multi-center research to validate GTOS in patients with TBI. Additionally, incorporating TBI-specific factors like intracranial pressure or brain injury types into GTOS may enhance its predictive accuracy. Long-term follow-up studies are also needed to evaluate GTOS’s effectiveness in predicting extended survival and functional recovery in TBI patients.

## 5. Conclusions

GTOS is a valuable tool for predicting in-hospital mortality in patients with isolated moderate to severe TBI, demonstrating significant accuracy. However, while GTOS provides a rapid screening method in clinical settings that can aid in risk stratification for these distinct patients, further research may be needed to refine GTOS for better applicability in TBI patients.

## Figures and Tables

**Table 1:** Demographic and clinical characteristics of patients stratified by in-hospital mortality status.

Variables	Death *n* = 462	Survival *n* = 5081	OR (95% CI)	*p*
Sex				0.006
Male, *n* (%)	312 (67.5)	3104 (61.1)	1.33 (1.08–1.62)	
Female, *n* (%)	150 (32.5)	1977 (38.9)	0.76 (0.62–0.92)	
Age, years (mean ± SD)	64.1 ± 18.2	58.0 ± 19.1	-	<0.001
PRBC, units	2.4 ± 4.4	0.4 ± 1.5	-	<0.001
GTOS	131.2 ± 25.5	100.5 ± 24.4	-	<0.001
Comorbidities				
CVA, *n* (%)	32 (6.9)	315 (6.2)	1.13 (0.77–1.64)	0.537
HTN, *n* (%)	198 (42.9)	1852 (36.4)	1.31 (1.08–1.59)	0.006
CAD, *n* (%)	58 (12.6)	320 (6.3)	2.14 (1.59–2.88)	<0.001
CHF, *n* (%)	4 (0.9)	39 (0.8)	1.13 (0.40–3.17)	0.818
DM, *n* (%)	93 (20.1)	1005 (19.8)	1.02 (0.81–1.30)	0.856
ESRD, *n* (%)	47 (10.2)	126 (2.5)	4.45 (3.14–6.32)	<0.001
GCS, median (IQR)	5 (3–11)	15 (13–15)	-	<0.001
3–8, *n* (%)	319 (69.0)	654 (12.9)	15.10 (12.20–18.70)	<0.001
9–12, *n* (%)	40 (8.7)	543 (10.7)	0.79 (0.57–1.11)	0.174
13–15, *n* (%)	103 (22.3)	3884 (76.4)	0.09 (0.07–0.11)	<0.001
ISS, median (IQR)	25 (16–25)	16 (13–20)	-	<0.001
1–15, *n* (%)	25 (5.4)	1669 (32.8)	0.12 (0.08–0.18)	<0.001
16–24, *n* (%)	128 (27.7)	2983 (58.7)	0.27 (0.22–0.33)	<0.001
≥25, *n* (%)	309 (66.9)	429 (8.4)	21.90 (17.62–27.22)	<0.001
Hospital stay (days)	9.3 ± 14.4	12.4 ± 12.9	-	<0.001

CAD, coronary artery disease; CHF, congestive heart failure; CVA, cerebrovascular accident; CI, confidence interval; DM, diabetes mellitus; ESRD, end-stage renal disease; GCS, Glasgow Coma Scale; GTOS, Geriatric Trauma Outcome Score; HTN, hypertension; IQR, interquartile range; ISS, injury severity score; OR, odds ratio; PRBC, packed red blood cells; SD, standard deviation.

**Table 2:** Univariate and multivariate analysis of factors associated with in-hospital mortality in isolated moderate to severe TBI patients.

Variables	Univariate Analysis			Multivariate Analysis		
	OR	CI	*p*	OR	CI	*p*
Male	1.33	(1.08–1.62)	0.007	1.46	(1.15–1.84)	0.002
Age	1.19	(1.13–1.26)	<0.001	0.98	(0.86–1.11)	0.734
GTOS	1.67	(1.59–1.75)	<0.001	1.33	(1.19–1.49)	<0.001
HTN	1.31	(1.08–1.59)	0.006	1.03	(0.80–1.31)	0.845
CAD	2.14	(1.59–2.88)	<0.001	1.42	(0.99–2.04)	0.054
ESRD	4.45	(3.14–6.32)	<0.001	4.72	(3.10–7.18)	<0.001
ISS	1.26	(1.24–1.29)	<0.001	1.17	(1.12–1.22)	<0.001

CAD, coronary artery disease; CI, confidence interval; ESRD, end-stage renal disease; GTOS, Geriatric Trauma Outcome Score; HTN, hypertension; ISS, injury severity score; OR, odds ratio.

**Table 3:** Variance inflation factor (VIF) of variables in the multivariate analysis of factors associated with in-hospital mortality in isolated moderate to severe TBI patients.

Model	Standardized Coefficients	t	*p*	Collinearity Statistics	
	Beta			Tolerance	VIF
(Constant)		35.634	0.000		
Male	0.031	2.447	0.014	0.974	1.027
Age	0.201	5.644	0.000	0.119	8.413
ISS	−0.098	−3.094	0.002	0.150	6.659
GTOS	−0.396	−8.793	0.000	0.075	13.396
HTN	−0.004	−318	0.750	0.759	1.317
CAD	0.030	2.343	0.019	0.927	1.079
ESRD	0.101	8.090	0.000	0.970	1.031

CAD, coronary artery disease; ESRD, end-stage renal disease; GTOS, Geriatric Trauma Outcome Score; HTN, hypertension; ISS, injury severity score; VIF, variance inflation factor.

**Table 4:** Comparative demographics and outcomes based on high (≥121.5) and low (<121.5) GTOS scores.

	GTOS			
	≥121.5 *n* = 1252	<121.5 *n* = 4291	OR (95% CI)	*p*
Sex				0.527
Male, *n* (%)	762 (60.9)	2654 (61.9)	0.96 (0.84–1.09)	
Female, *n* (%)	490 (39.1)	1637 (38.1)	1.04 (0.92–1.19)	
Age, years (mean ± SD)	73.6 ± 13.6	54.1 ± 18.2	-	<0.001
PRBC, units	1.8 ± 3.2	0.2 ± 1.2	-	<0.001
Comorbidities				
CVA, *n* (%)	120 (9.6)	227 (5.3)	1.90 (1.51–2.39)	<0.001
HTN, *n* (%)	658 (52.6)	1392 (32.4)	2.31 (2.03–2.62)	<0.001
CAD, *n* (%)	169 (13.5)	209 (4.9)	3.05 (2.46–3.77)	<0.001
CHF, *n* (%)	16 (1.3)	27 (0.6)	2.04 (1.10–3.81)	0.021
DM, *n* (%)	310 (24.8)	788 (18.4)	1.46 (1.26–1.70)	<0.001
ESRD, *n* (%)	59 (4.7)	114 (2.7)	1.81 (1.32–2.50)	<0.001
GCS, median (IQR)	13 (6–15)	15 (13–15)	-	<0.001
3–8, *n* (%)	433 (34.6)	540 (12.6)	3.67 (3.17–4.26)	<0.001
9–12, *n* (%)	155 (12.4)	428 (10.0)	1.28 (1.05–1.55)	0.015
13–15, *n* (%)	664 (53.0)	3323 (77.4)	0.33 (0.29–0.38)	<0.001
ISS, median (IQR)	24 (16–25)	16 (10–17)	-	<0.001
1–15, *n* (%)	27 (2.2)	1667 (38.8)	0.04 (0.02–0.05)	<0.001
16–24, *n* (%)	661 (52.8)	2450 (57.1)	0.84 (0.74–0.95)	0.007
≥25, *n* (%)	564 (45.0)	174 (4.1)	19.40 (16.07–23.41)	<0.001
Mortality, *n* (%)	315 (25.2)	147 (3.4)	9.48 (7.70–11.67)	<0.001
Hospital stay (days)	15.7 ± 16.5	11.0 ± 11.7	-	<0.001

CAD, coronary artery disease; CHF, congestive heart failure; CVA, cerebrovascular accident; CI, confidence interval; DM, diabetes mellitus; ESRD, end-stage renal disease; GCS, Glasgow Coma Scale; GTOS, Geriatric Trauma Outcome Score; HTN, hypertension; IQR, interquartile range; ISS, injury severity score; OR, odds ratio; PRBC, packed red blood cells; SD, standard deviation.

**Table 5:** Propensity-score-matched analysis of high (≥121.5) and low (<121.5) GTOS score groups.

Propensity Score Matched-Cohort								
	GTOS							
≥121.5 *n* = 794		<121.5 *n* = 794		OR (95% CI)			*p*	SD
Sex								
Male, *n* (%)	492	(62.0)	492	(62.0)	1.00	(0.82–1.23)	1.000	0.00%
Age, years ± SD	69.9	±14.1	69.2	±13.6	-		0.292	5.29%
Co-Morbidity								
CVA, *n* (%)	61	(7.7)	61	(7.7)	1.00	(0.69–1.45)	1.000	0.00%
HTN, *n* (%)	389	(49.0)	389	(49.0)	1.00	(0.82–1.22)	1.000	0.00%
CAD, *n* (%)	78	(9.8)	78	(9.8)	1.00	(0.72–1.39)	1.000	0.00%
CHF, *n* (%)	2	(0.3)	2	(0.3)	1.00	(0.14–7.12)	1.000	0.00%
DM, *n* (%)	189	(23.8)	189	(23.8)	1.00	(0.79–1.26)	1.000	0.00%
ESRD, *n* (%)	20	(2.5)	20	(2.5)	1.00	(0.53–1.87)	1.000	0.00%
GCS, median (IQR)	15	(9–15)	14	(14–15)	-		0.353	4.66%
Mortality, *n* (%)	147	(18.5)	63	(7.9)	2.64	(1.93–3.61)	<0.001	-
Hospital stay (days)	16.7	±16.2	12.2	±12.8	-		<0.001	-

CAD, coronary artery disease; CHF, congestive heart failure; CVA, cerebrovascular accident; CI, confidence interval; DM, diabetes mellitus; ESRD, end-stage renal disease; GCS, Glasgow Coma Scale; GTOS, Geriatric Trauma Outcome Score; HTN, hypertension; IQR, interquartile range; OR, odds ratio; SD, standard deviation.

## Data Availability

The original contributions presented in the study are included in the article, further inquiries can be directed to the corresponding author.

## References

[1] Harrison-Felix C.L., Whiteneck G.G., Jha A., De Vivo M.J., Hammond F.M., Hart D.M. (2009). Mortality over four decades after traumatic brain injury rehabilitation: A retrospective cohort study. Arch. Phys. Med. Rehabil..

[2] Greenwald B.D., Hammond F.M., Harrison-Felix C., Nakase-Richardson R., Howe L.L., Kreider S. (2015). Mortality following Traumatic Brain Injury among Individuals Unable to Follow Commands at the Time of Rehabilitation Admission: A National Institute on Disability and Rehabilitation Research Traumatic Brain Injury Model Systems Study. J. Neurotrauma.

[3] Utomo W.K., Gabbe B.J., Simpson P.M., Cameron P.A. (2009). Predictors of in-hospital mortality and 6-month functional outcomes in older adults after moderate to severe traumatic brain injury. Injury.

[4] Ahl R., Phelan H.A., Dogan S., Cao Y., Cook A.C., Mohseni S. (2017). Predicting In-Hospital and 1-Year Mortality in Geriatric Trauma Patients Using Geriatric Trauma Outcome Score. J. Am. Coll. Surg..

[5] Ravindranath S., Ho K.M., Rao S., Nasim S., Burrell M. (2021). Validation of the geriatric trauma outcome scores in predicting outcomes of elderly trauma patients. Injury.

[6] Scherer J., Kalbas Y., Ziegenhain F., Neuhaus V., Lefering R., Teuben M., Sprengel K., Pape H.C., Jensen K.O. (2021). The GERtality Score: The Development of a Simple Tool to Help Predict in-Hospital Mortality in Geriatric Trauma Patients. J. Clin. Med..

[7] Zhao F.Z., Wolf S.E., Nakonezny P.A., Minhajuddin A., Rhodes R.L., Paulk M.E., Phelan H.A. (2015). Estimating Geriatric Mortality after Injury Using Age, Injury Severity, and Performance of a Transfusion: The Geriatric Trauma Outcome Score. J. Palliat. Med..

[8] Zhuang Y., Feng Q., Tang H., Wang Y., Li Z., Bai X. (2022). Predictive Value of the Geriatric Trauma Outcome Score in Older Patients After Trauma: A Retrospective Cohort Study. Int. J. Gen. Med..

[9] Cook A.C., Joseph B., Inaba K., Nakonezny P.A., Bruns B.R., Kerby J.D., Brasel K.J., Wolf S.E., Cuschieri J., Paulk M.E. (2016). Multicenter external validation of the Geriatric Trauma Outcome Score: A study by the Prognostic Assessment of Life and Limitations After Trauma in the Elderly (PALLIATE) consortium. J. Trauma Acute Care Surg..

[10] Arslan Erduhan M., Doğan H., Ilhan B. (2023). Relationships of the frailty index and geriatric trauma outcome score with mortality in geriatric trauma patients. Ulus. Travma Acil Cerrahi Derg..

[11] Meagher A.D., Lin A., Mandell S.P., Bulger E., Newgard C. (2019). A Comparison of Scoring Systems for Predicting Short- and Long-term Survival After Trauma in Older Adults. Acad. Emerg. Med..

[12] Madni T.D., Ekeh A.P., Brakenridge S.C., Brasel K.J., Joseph B., Inaba K., Bruns B.R., Kerby J.D., Cuschieri J., Mohler M.J. (2017). A comparison of prognosis calculators for geriatric trauma: A Prognostic Assessment of Life and Limitations After Trauma in the Elderly consortium study. J. Trauma Acute Care Surg..

[13] Park J., Lee Y. (2022). Predicting Mortality of Korean Geriatric Trauma Patients: A Comparison between Geriatric Trauma Outcome Score and Trauma and Injury Severity Score. Yonsei Med. J..

[14] Moorthy D., Rajesh K., Priya S.M., Abhinov T., Devendra Prasad K.J. (2021). Prediction of Outcome Based on Trauma and Injury Severity Score, IMPACT and CRASH Prognostic Models in Moderate-to-Severe Traumatic Brain Injury in the Elderly. Asian J. Neurosurg..

[15] Knaus W.A., Draper E.A., Wagner D.P., Zimmerman J.E. (1985). APACHE II: A severity of disease classification system. Crit. Care Med..

[16] Le Gall J.R., Lemeshow S., Saulnier F. (1993). A new Simplified Acute Physiology Score (SAPS II) based on a European/North American multicenter study. JAMA.

[17] Lemeshow S., Teres D., Klar J., Avrunin J.S., Gehlbach S.H., Rapoport J. (1993). Mortality Probability Models (MPM II) based on an international cohort of intensive care unit patients. JAMA.

[18] Vincent J.L., Moreno R., Takala J., Willatts S., De Mendonça A., Bruining H., Reinhart C.K., Suter P.M., Thijs L.G. (1996). The SOFA (Sepsis-related Organ Failure Assessment) score to describe organ dysfunction/failure: On behalf of the Working Group on Sepsis-Related Problems of the European Society of Intensive Care Medicine. Intensive Care Med..

[19] Tsai C.H., Liu H.T., Hsieh T.M., Huang C.Y., Chou S.E., Su W.T., Li C., Hsu S.Y., Hsieh C.H. (2022). Change of neutrophil-to-monocyte ratio to stratify the mortality risk of adult patients with trauma in the intensive care units. Formos. J. Surg..

[20] Fluss R., Faraggi D., Reiser B. (2005). Estimation of the Youden Index and its associated cutoff point. Biom. J..

[21] Perkins N.J., Schisterman E.F. (2005). The Youden Index and the optimal cut-point corrected for measurement error. Biom. J..

[22] Salim A., Hadjizacharia P., Du Bose J., Brown C., Inaba K., Chan L., Margulies D.R. (2008). Role of anemia in traumatic brain injury. J. Am. Coll. Surg..

[23] Warner M.A., O’Keeffe T., Bhavsar P., Shringer R., Moore C., Harper C., Madden C.J., Sarode R., Gentilello L.M., Diaz-Arrastia R. (2010). Transfusions and long-term functional outcomes in traumatic brain injury. J. Neurosurg..

[24] Acker S.N., Partrick D.A., Ross J.T., Nadlonek N.A., Bronsert M., Bensard D.D. (2014). Blood component transfusion increases the risk of death in children with traumatic brain injury. J. Trauma Acute Care Surg..

[25] Litofsky N.S., Martin S., Diaz J., Ge B., Petroski G., Miller D.C., Barnes S.L. (2016). The Negative Impact of Anemia in Outcome from Traumatic Brain Injury. World Neurosurg..

[26] Benson C., Aminoff M.J., Daroff R.B. (2014). Diffuse Axonal Injury. Encyclopedia of the Neurological Sciences.

[27] Huh J.W., Raghupathi R. (2007). Chronic Cognitive Deficits and Long-Term Histopathological Alterations following Contusive Brain Injury in the Immature Rat. J. Neurotrauma.

[28] Smith M. (2003). Diffuse axonal injury in adults. Trauma.

[29] Mingℯ H.K., Ravindranath S. (2022). Geriatric Trauma Outcome Score (GTOS) in elderly patients with traumatic brain injury. Injury.

[30] Barea-Mendoza J.A., Chico-Fernández M., Sánchez-Casado M., Llompart-Pou J.A. (2022). Performance of the Geriatric Trauma Outcome Score in traumatic brain injury: A call of caution. Injury.

[31] Christie S.A., Conroy A.S., Callcut R.A., Hubbard A.E., Cohen M.J. (2019). Dynamic multi-outcome prediction after injury: Applying adaptive machine learning for precision medicine in trauma. PLoS ONE.

[32] Rau C.-S., Kuo P.-J., Chien P.-C., Huang C.-Y., Hsieh H.-Y., Hsieh C.-H. (2018). Mortality prediction in patients with isolated moderate and severe traumatic brain injury using machine learning models. PLoS ONE.

